# Sex-Specific Differences in Lower Body Fat Distribution and Association with Physical Performance among Healthy Community-Dwelling Older Adults: A Pilot Study

**DOI:** 10.3390/ijerph19074201

**Published:** 2022-04-01

**Authors:** Miji Kim, Jang-Hoon Oh, Chang Won Won

**Affiliations:** 1 Department of Biomedical Science and Technology, College of Medicine, East-West Medical Research Institute, Kyung Hee University, Seoul 02447, Korea; 2 Department of Biomedical Science and Technology, Graduate School, Kyung Hee University, Seoul 02447, Korea; roineri5@gmail.com; 3 Department of Family Medicine, College of Medicine, Kyung Hee University, Seoul 02447, Korea

**Keywords:** body composition, physical performance, adiposity

## Abstract

This study aims to examine sex-specific differences in body composition and lower extremity fat distribution and their association with physical performance among healthy older adults. The pilot study comprises 40 subjects (20 men and 20 women) matched by age and body mass index. The participants undergo dual-energy X-ray absorptiometry, magnetic resonance imaging, and proton magnetic resonance spectroscopy (^1^H-MRS) to assess body composition and lower extremity fat distribution. ^1^H-MRS is used to measure the extramyocellular lipid (EMCL) and intramyocellular lipid (IMCL) contents of the lower leg muscles (soleus and tibialis anterior) at the maximum circumference of the calf after overnight fasting. The tibialis anterior IMCL, as assessed by ^1^H-MRS, is negatively associated with the five-times sit-to-stand test scores (r_s_ = 0.518, *p* = 0.023) in men, while the soleus IMCL content is negatively associated with the timed up-and-go test scores (r_s_ = 0.472, *p* = 0.048) in women. However, the soleus EMCL content is positively associated with the five-times sit-to-stand test scores (r_s_ = −0.488, *p* = 0.040) in women, but this association is not statistically significant in men. This study shows an inverse correlation between IMCL content and physical performance in healthy older individuals and lower leg muscle-specific IMCL based on sex differences. Furthermore, our results suggest that greater EMCL content in the soleus and calf subcutaneous fat might affect physical performance positively in women but not men.

## 1. Introduction

Physical disabilities and limitations in lower extremity function are common with increasing age [1]. Physical performance tests for lower extremity function are significant clinical predictors of incident falls, disability, hospitalization, and mortality in older adults [2,3,4,5,6]. Furthermore, physical performance has been used to assess frailty and is one of the most important screening components and diagnostic criterion for sarcopenia. Identifying sarcopenia severity in older adults is also important [7,8,9].

Sex-specific differences in physical performance among older adults have been observed in several studies [1,10,11,12,13,14]; a previous study reported that physical performance in older adults continually declines with increasing age, with sex-specific differences [15]. These studies demonstrated that older women have poorer physical performance and higher mobility limitations than older men. Studies to date reported that sex-specific differences in lower extremity performance might be related to fat mass, muscle fat infiltration, and muscle mass [16,17,18,19]; there are differences in body composition between men and women, with women having a higher body fat mass and lower muscle mass than men. Therefore, considering the various aspects of body composition with respect to sex-specific differences is important in age research.

The inter-sex difference in fat distribution is a heterogeneous phenotype, i.e., women have greater amounts of intramuscular fat than men [20]. Regarding lower extremity fat distribution in adults, young women have greater intramyocellular lipid (IMCL) content in the soleus muscle than young men, and IMCL exists as droplets near the mitochondria of skeletal muscle cells based on proton magnetic resonance spectroscopy (^1^H-MRS) [21]. In vivo ^1^H-MRS can noninvasively assess the amount of extramyocellular lipid (EMCL) and IMCL in humans [22,23]. Recent evidence suggests that skeletal muscle fiber-type composition is dependent on anatomical location, function, and sex [24,25]. The tibialis anterior contains fast muscle fibers, whereas the soleus is mainly composed of slow muscle fibers, as observed by q-space imaging [26]. In a study of human muscle fiber-type composition, men were found to have more fast muscle fibers, and larger muscle fibers, than women [27]. A previous study suggested that the intramuscular lipid content in the quadriceps muscle may have sex-specific effects on whole-muscle and cellular contractile functions in older adults, with stronger associations in women [28].

To the best of our knowledge, the association between ectopic fat, such as lower-extremity fat distribution, sex-specific differences, and poor physical performance among healthy older adults remains to be determined. Understanding the role of muscle-specific fat infiltration based on sex differences and its relationship to physical performance in community-dwelling older adults has important public health implications. Therefore, in this preliminary study, we examine sex-specific differences in the whole body as well as regional body composition and fat distribution in the soleus and tibialis anterior muscles noninvasively using quantitative methods for muscle fat infiltration in vivo using ^1^H-MRS. We also study their association with physical performance among healthy older men and women matched by age and body mass index (BMI).

## 2. Materials and Methods

### 2.1. Subjects

The subjects were randomly selected from the Korean Frailty and Aging Cohort Study (KFACS) and matched by age and BMI. The KFACS is an ongoing multicenter longitudinal cohort study that recruited community-dwelling individuals aged 70–84 years from 10 centers. The baseline survey was conducted in 2016–2017, with each site contributed approximately 300 participants [29]. The pilot study group comprised 40 participants (20 men, 20 women) recruited in the second wave of the survey (2018–2019) from Kyung Hee University Medical Center. The sample size for our pilot study was set according to that in previous studies that used an exploratory case study of fat distribution among older adults [30,31]. The exclusion criteria were: diseases that affect fat distribution and/or physical performance [32,33,34]; a clinical history of diabetes mellitus, heart disease (myocardial infarction, congestive heart failure, and angina pectoris), cerebrovascular disease (stroke, cerebral infarction, and cerebral hemorrhage), Parkinson’s disease, or previous hip and knee replacements; and patients with contraindications for magnetic resonance imaging (MRI). The participants underwent dual-energy X-ray absorptiometry (DXA), MRI, and ^1^H-MRS to assess their body fat distribution and body composition.

The study protocol was approved by the Clinical Research Ethics Committee of Kyung Hee University Hospital (no. 2017-12-015-002). All participants were given prior explanations and signed consent forms.

### 2.2. Dual-Energy X-ray Absorptiometry

DXA (Lunar iDXA; GE Healthcare, Madison, WI, USA) was used to measure whole and regional body composition. The participants were positioned for whole-body scans according to the manufacturer’s protocol. Before the scan, the participants were asked to remove all metal accessories. They laid down in a supine position on the scanner table with their limbs close to their bodies. The arm, leg, and trunk segments were separated manually by anatomical landmarks using the DXA analysis software (enCORE, version 11). Total and segmental body compositions such as fat mass (kg) and lean mass (kg) were calculated.

### 2.3. Magnetic Resonance Imaging

A three-Tesla MRI scanner was used to acquire images of the right lower leg (Achieva; Philips Medical Systems, Best, The Netherlands). A two-dimensional sagittal fast spin echo (FSE) localizer was used to position the participant’s leg in the system, and 20 axial T2-weighted turbo spin-echo images were obtained. Transverse images were obtained from the distal end of the medial malleolus to the medial knee joint cleft using the following parameters: repetition time, 1982 ms; echo time, 100 ms; flip angle, 90°; matrix size, 560 × 560; and field of view, 162 × 162 (pixel size) and 0.29 × 0.29 mm^2^. In all calf image slices of single individuals, the single slice with the largest area was selected for cross-sectional area (CSA, cm^2^) measurements. A selected calf image was loaded into SliceOmatic (4.3 rev. 8b; Tomovision, Montreal, QC, Canada) to obtain the CSA measurements. Gamma correction processing was applied to all images to calibrate the gray-level intensity of the images for slice-by-slice segmentation. CSA was segmented automatically first by the combination of functions offered by the software, including threshold, region growing, mathematical morphology, and active contours, and the segment labels from the software were manually modified by humans. We segmented the compartments of the lower leg according to the following definitions based on a previous study [35]: (1) subcutaneous adipose tissue: any tissue or component outside the fascia, including vessels and skin; (2) total muscle: non-adipose tissue beneath the fascia, excluding bone; (3) intermuscular fat: adipose tissue beneath the fascia; and (4) intramuscular fat: adipose tissue within the total muscle compartment, including vessels. All scans were read by the same technician, who was blinded to the scan sequence.

### 2.4. Proton Magnetic Resonance Spectroscopy

^1^H-MRS was used to measure the EMCL and IMCL content of the lower leg (soleus and tibialis anterior) muscles at the maximum circumference of the calf after overnight fasting [36]. We used a three-Tesla magnetic resonance device (Achieva; Philips Medical System, The Netherlands) equipped with a 32-channel SENSE cardiac coil to acquire MRS data using the point-resolved spectroscopy (PRESS) sequence. The PRESS sequence parameters were as follows: repetition time, 2000 ms; echo time, 50 ms; bandwidth, 2000 Hz; 2048 sampling points; average of 96 (with and without water suppression); and volume of interest (VOI), 20 mm × 20 mm × 20 mm.

The data obtained were analyzed using LCModel software (version 6.3-1N; Stephen Provencher, Oakville, ON, Canada). The concentrations of IMCL-CH_2_ (1.3 ppm) and EMCL-CH_2_ (1.5 ppm) were computed as mmol (mM) per liter of muscle tissue with an unsuppressed water peak. To convert mM to mmol/kg wet weight, the value was divided by the tissue density (1.05 kg/L for normal muscle tissue) [37]. In the analysis results, the standard deviation was less than 20%, which is considered reliable for assessing EMCL or IMCL content in the muscles [36]. All scans were read by the same technician, who was blinded to the scan sequence.

### 2.5. Muscle Strength and Physical Performance Measurement

Handgrip strength was measured using a digital handgrip dynamometer (T.K.K.5401; Takei Scientific Instruments Co., Ltd., Tokyo, Japan). The participants stood upright with the shoulders in a neutral position, arms at the sides, and elbows fully extended. The participants were instructed to squeeze the handle with maximum effort for 3 s using each hand while receiving verbal encouragement. Each hand was tested twice, with alternating hands between trials and a 3-min rest period between measurements of the same hand. The highest measurement of each hand was recorded as the maximum handgrip strength expressed in kilograms [38].

Physical performance was evaluated using the usual gait speed, five-times sit-to-stand test, Short Physical Performance Battery (SPPB), and timed up-and-go (TUG) test. The usual gait speed over a 4-m distance was measured using an automatic gait speed meter (Dynamicphysiology, Daejeon, Korea) with acceleration and deceleration phases of 1.5 m each. Participants were asked to perform the test by walking at a normal pace. The participants performed two trials and the results were averaged to the nearest 0.01 m/s. The five-times sit-to-stand test measures the time taken to stand five times from a sitting position without using the arms from a straight-backed armchair. The participants were asked to stand up and sit down five times as quickly as possible. The time from the initial sitting position when the examiner said “go” to the final fully erect position at the end of the fifth stand was measured to the nearest 0.01 s [39]. The SPPB consists of three standing balance measures, the five-times sit-to-stand test scores, and an assessment of usual gait speed [39]. Each test is assigned a score of 0–4 based on the normative scores obtained from the Established Population for Epidemiologic Studies of the Elderly, with a total possible score of 0–12 [1]. The TUG test measures the time taken to walk a distance of 3 m at a comfortable pace around an obstacle and return to sitting on a chair [40].

### 2.6. Anthropometric Measurements

Anthropometric indicators included height, weight, BMI, waist circumference, hip circumference, right mid-thigh circumference, right calf circumference, and waist-to-hip ratio. Height and body weight were measured to the nearest 0.1 cm or 0.1 kg, respectively, while subjects were clothed in light garments. Patient BMI was calculated as weight divided by height squared (kg/m^2^). Waist circumference was measured to the nearest 0.1 cm at the midpoint between the bottom of the rib cage and the top of the lateral border of the iliac crest during minimal respiration, and hip circumference was defined as the greatest circumference between the hips and buttocks. The waist-to-hip ratio was calculated as the waist circumference divided by the hip circumference. Mid-thigh circumference was measured using the right thigh at the midpoint between the inguinal crease and proximal border of the patella. Calf circumference was measured as the maximum horizontal distance around the right calf while participants stood upright.

### 2.7. Blood Biochemical Analysis

Blood samples were drawn from the antecubital vein after an overnight fast of ≥8 h. The participants did not take any medications before the blood samples were collected. Blood samples were immediately stored at low temperatures (2–8 °C) and transported to central clinical laboratories (Seegene Medical Foundation, Seoul, Korea) within 24 h of sample collection. All biological samples were stored at −80 °C before analysis. Biochemical measurements included those of serum insulin, glucose, glycosylated hemoglobin, total cholesterol, high-density lipoprotein cholesterol, low-density lipoprotein cholesterol, triglycerides, albumin, creatinine, 25-hydroxyvitamin D, and high-sensitivity C-reactive protein.

### 2.8. Statistical Analysis

Data are presented as median (interquartile range) or number (percentage) for both sexes. We used the Shapiro–Wilk test to assess normality. The Mann–Whitney U test was used to assess continuous variables between the groups divided based on sex, as the data were not normally distributed. Categorical variables were compared using chi-square or Fisher exact tests. The relationship between body composition and physical performance in men and women was evaluated using Spearman’s rank correlation coefficients (r_s_). The statistical analyses and graphical representations were performed using IBM SPSS Statistics for Windows (version 26.0; IBM Corp., Armonk, NY, USA) and R software (version 3.6.2; R Core Team, Vienna, Austria). Statistical significance was set at *p* < 0.05.

## 3. Results

### 3.1. Clinical Characteristics of the Subjects

The clinical characteristics of our study cohort stratified by sex are shown in Table 1. The age difference between the men and women was not statistically significant (*p* = 0.072). Men had a greater height and weight than women, resulting in them having similar median BMIs (median, 24.1 kg/m^2^ vs. 24.2 kg/m^2^; *p* = 0.841). Compared with women, men had a significantly median greater waist circumference, waist-to-hip ratio, and calf circumference. However, there were no significant inter-sexes difference in the median hip and thigh circumferences. Men and women had similar physical activity levels and chronic health conditions (*p* > 0.05). There were no significant differences in any biochemical parameters between the blood samples of men and women except for high-density lipoprotein cholesterol, creatinine, and albumin. Grip strength was significantly higher in men than in women (median, 34.4 kg vs. 19.6 kg; *p* = 0.001). However, there were no significant inter-sex differences in the other physical performance test results (all *p* > 0.05).

### 3.2. Sex-Specific Differences in Body Composition

**Figure 1:**
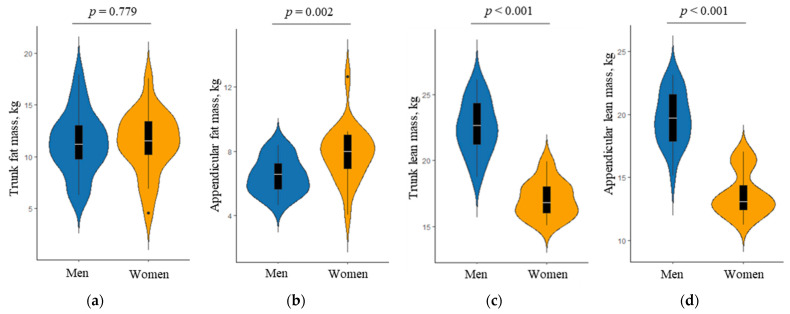
Comparison of segmental body composition as assessed by dual-energy X-ray absorptiometry between men and women: (**a**) trunk fat mass, (**b**) appendicular fat mass, (**c**) trunk lean mass, and (**d**) appendicular lean mass. The *p* value was obtained by the Mann–Whitney U test. Violin plots show the distribution of body composition in men and women. Overlaid box plots show medians, interquartile ranges, and spikes extending to the upper- and lower-adjacent values in each group.

Sex-specific differences in segmental body composition, as measured by DXA, are shown in Figure 1. There was no significant inter-sex difference in the median trunk fat mass. However, women had a greater appendicular fat mass than men (median, 8.0 kg vs. 6.6 kg; *p* = 0.002), while men had greater trunk and appendicular lean masses. The whole-body composition measures were similar to those of the segmental body composition (data not shown). There was no significant difference in the median total fat mass between men and women (median, 17.9 kg vs. 20.2 kg; *p* = 265). The median total lean mass was greater in women than in men (median, 45.4 kg vs. 33.0 kg; *p* < 0.001). However, women had a greater body fat percentage, being the total fat mass divided by total body mass, than men (median, 36.5% vs. 28.0%; *p* < 0.001).

**Figure 2:**
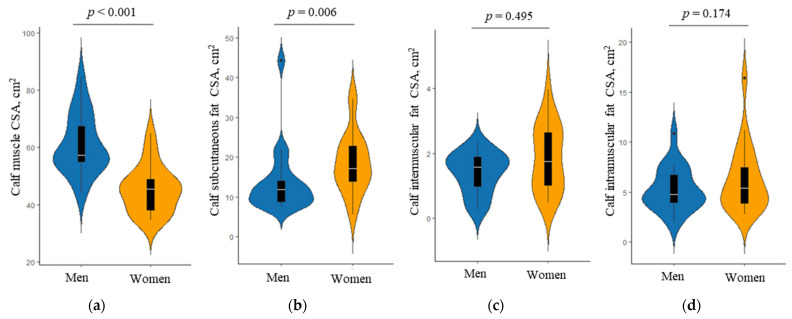
Comparison of the calf cross-sectional area (CSA) as assessed by magnetic resonance imaging between men and women; (**a**) calf muscle CSA, (**b**) calf subcutaneous fat CSA, (**c**) calf intermuscular fat CSA, and (**d**) calf intramuscular fat CSA. The *p* value was obtained by the Mann–Whitney U test. Violin plots show the distribution of body composition in men and women. Overlaid box plots show medians, interquartile ranges, and spikes extending to the upper- and lower-adjacent values in each group.

Sex-specific differences in the calf CSA, as assessed by MRI, are shown in Figure 2. The median calf muscle CSA was significantly greater in women than in men (median, 57.2 cm^2^ vs. 45.4 cm^2^; *p* < 0.001). The calf subcutaneous fat CSA was greater in women than in men (median, 17.1 cm^2^ vs. 11.8 cm^2^; *p* = 0.006). However, the calf intermuscular and intramuscular fat CSA did not differ significantly between them (all *p* > 0.05).

**Figure 3:**
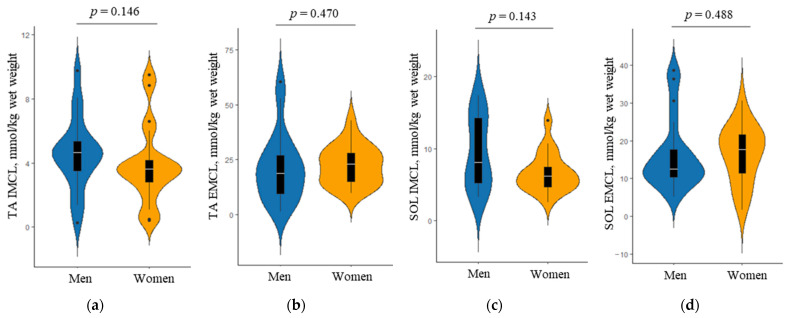
Comparison of the amounts of intramyocellular lipid (IMCL) and extramyocellular lipid (EMCL) in the tibialis anterior (TA) and soleus (SOL), as assessed by proton magnetic resonance spectroscopy, between men and women; (**a**) TA IMCL, (**b**) TA EMCL, (**c**) SOL IMCL, and (**d**) SOL EMCL. The *p* value was obtained by the Mann–Whitney U test. Violin plots show the distribution of body composition in men and women. Overlaid box plots show medians, interquartile ranges, and spikes extending to the upper- and lower-adjacent values in each group.

Sex-specific differences in the IMCL and EMCL of the tibialis anterior and soleus, as assessed by ^1^H-MRS, are shown in Figure 3. There was no significant difference in the median amounts of both IMCL and EMCL between men and women (all *p* > 0.05). However, the violin plots showed that the trend in the IMCL content in the tibialis anterior and soleus was greater in men than in women. Additionally, this trend was also observed in the EMCL content in both muscles.

### 3.3. Differences in the Association of Body Composition with Physical Performance between Men and Women

The sex-specific differences in the association between body composition and physical performance are shown in Table 2 and Table 3. Regarding the body composition as measured by DXA, total lean mass (men, r_s_ = 0.498, *p* = 0.026; women, r_s_ = 0.467, *p* = 0.038) and appendicular lean mass (men, r_s_ = 0.568, *p* = 0.007; women, r_s_ = 0.505, *p* = 0.023) were positively correlated with grip strength. Body fat percentage was negatively associated with grip strength (r_s_ = −0.483, *p* = 0.031) in men but not in women. Body fat percentage (r_s_ = 0.470, *p* = 0.036) and right leg fat mass (r_s_ = 0.462, *p* = 0.040) were positively correlated with the SPPB scores in women, but this association was not observed in men. 

Regarding the calf CSA parameters assessed by MRI, the calf intermuscular fat CSA was positively correlated with the five-times sit-to-stand test scores in men (r_s_ = −0.553, *p* = 0.011). Furthermore, the calf intramuscular fat CSA had a significant negative correlation with the five-times sit-to-stand test scores in men. The calf muscle CSA tended to be positively associated with grip strength (r_s_ = 0.417, *p* = 0.068) and the TUG test scores (r_s_ = −0.415, *p* = 0.069) in men but not in women. In contrast, calf subcutaneous fat CSA was positively associated with the usual gait speed (r_s_ = 0.447, *p* = 0.048) in women only. Furthermore, calf subcutaneous fat CSA tended to be positively associated with the five-times sit-to-stand test, TUG test, and SPPB scores in women (all *p* < 0.10), but these associations were not statistically significant in men.

Regarding the IMCL and EMCL parameters of the tibialis anterior and soleus muscles as assessed by ^1^H-MRS, the tibialis anterior IMCL content was negatively associated with the five-times sit-to-stand test scores (r_s_ = 0.518, *p* = 0.023) in men. The soleus IMCL content was negatively associated with the TUG test scores (r_s_ = 0.472, *p* = 0.048) in women. However, the soleus EMCL content was positively associated with the five-times sit-to-stand test scores (r_s_ = −0.488, *p* = 0.040) in women, but this association was not statistically significant in men. Furthermore, the tibialis anterior EMCL content tended to be positively associated with grip strength in women only (r_s_ = 0.433, *p* = 0.064).

## 4. Discussion

The findings of this pilot study indicate that in healthy older adults, muscle-specific calf IMCL content in men and women was negatively associated with physical performance; men had a greater IMCL content in the tibialis anterior, while women had a greater IMCL content in the soleus, which were associated with poor physical performance. Furthermore, a relatively greater EMCL content in the soleus was positively associated with physical performance in women but not in men. We also found that calf subcutaneous fat CSA was positively associated with gait speed in women; however, this association was not observed in men.

Our study demonstrated an inverse correlation between the amount of IMCL and physical performance in healthy older adults. Ectopic fat deposition, fat redistribution, amount of intermuscular and intramuscular adipose tissues, and IMCL content tend to increase with advancing age, while the amount of subcutaneous fat decreases [30,41,42,43]. IMCL is a lipid species that exists as droplets within myocytes and plays an important role in energy metabolism in the muscle cells [41]. ^1^H MRS techniques can be used to specifically quantify IMCL as lipid droplets both noninvasively and in vivo [22,23]. In older populations, a higher IMCL content in skeletal muscles responds to metabolic derangements and is associated with reduced mitochondrial adenosine triphosphate (ATP) production and the development of insulin resistance [44,45]. Crane et al. reported that reductions in the association of larger IMCL droplets with fewer mitochondria occurred with aging [46]. Furthermore, skeletal muscle ATP and IMCL are strongly associated with physical performance in older adults [47]. Correspondingly, our data suggest that higher IMCL content in the skeletal muscles of the lower leg may be explained by declines in physical performance that occur with aging. Interestingly, our study demonstrated that muscle-specific IMCL content in the lower leg is associated with physical performance and shows sex-specific differences. In men, a greater IMCL content in the tibialis anterior was associated with poor physical performance, but women had a greater IMCL content in the soleus. One possible explanation is the sex-specific differences in muscle fiber-type composition. The tibialis anterior contains fast muscle fibers, while the soleus is mainly composed of slow muscle fibers, as observed by q-space imaging [26]. The soleus intermedius muscles have a 70% higher proportion of slow twitch fibers than other leg muscles (gastrocnemius, vastus lateralis, and intermedius) in humans [48]. There are sex-specific differences in the skeletal muscle fiber-type composition. There is a greater prevalence of slower type-I and type-IIA fibers in women than in men, which parallels the lower contractile velocity in women than in men [24].

To explain sex-specific differences in skeletal muscle performance and fiber-type composition, the effects of variations in thyroid hormones, estrogen, and testosterone levels should also be considered [24]. Thyroid hormones are major determinants of muscle fiber composition. Individuals with hypothyroidism show characteristic myopathy, especially affecting type II muscle fibers [49]. Additionally, hypothyroid conditions are associated with the conversion of fast fiber types to slow fiber types [50]. However, McKeran et al. reported that type II fiber atrophy occurs in women with hypothyroid conditions but not in men with hypothyroid conditions [51]. Furthermore, an animal study demonstrated that the estrogen-induced increase in preproenkephalin mRNA levels in the ventromedial hypothalamus was reduced by the co-administration of triiodothyronine in hypophysectomized female rats [52]. These results suggest that thyroid hormone and estrogen receptors may interact to modulate estrogen-sensitive gene expression. Regarding the influence of estrogen on muscle fibers, it has been suggested that estrogen induces minimal changes in fiber-type distribution, with sex-specific differences [24]. Testosterone is associated with an increase in muscle mass, and testosterone deficiency leads to a decrease in fast-twitch fiber diameter, conversion to slow-twitch fibers, and enhanced fatigue resistance in men, but not in women [24].

Our study showed that a greater EMCL content in the soleus was positively associated with physical performance in the five-times sit-to-stand test in women but not men. EMCL, which exists in the stroma and as lipid depots in the skeletal muscles, is located in interstitial adipose tissues [23]. The EMCL content was positively associated with abdominal visceral fat, subcutaneous fat, and upper and lower limb fat content in participants aged 18–81 years. The role of the muscle EMCL in physical performance remains unclear. Zhu et al. suggested that the EMCL of the skeletal muscle tissue may serve as a hydrocarbon source for maintaining stability [53]. Prolonged moderate-intensity exercise reduced IMCL content by 27–56% in the soleus, while EMCL content in the adipocytes did not change during muscular contractions [54]. In this study, calf subcutaneous fat CSA was positively associated with physical performance in women but not in men. Subcutaneous adipose tissue plays a major metabolic role in regulating lipid energy storage and mobilization [55]. Adipose tissue is the main source of extra-glandular estrogen synthesis in postmenopausal women [56,57]. A recent review suggested that leptin and adiponectin levels are positively associated with the lower body subcutaneous adipose tissue while the level of inflammatory cytokines is negatively associated [58]. Therefore, greater EMCL content in the soleus and calf subcutaneous fat might affect physical performance in older women. Future studies are needed to investigate the underlying role of muscle EMCL in the physical performance in older adults.

In the present study, the total and appendicular lean masses were positively correlated with grip strength in both sexes, similar to the findings of previous studies [59,60]. In addition, our study showed that body fat percentage was negatively associated with grip strength in men but not in women. Body fat percentage and right leg fat mass were positively correlated with the SPPB scores in women, but this association was not observed in men. In contrast to our results, the Health, Aging, and Body Composition study reported that poorer physical performance in older women versus men can be explained predominantly by higher body fat mass in women [17]. It was also shown that body fat percentage was associated with poor physical function, while appendicular lean mass was associated with better physical function in older adult participants with various comorbidities, and there was no significant discrepancy by sex [61]. In several studies, the association between high body fat and physical disability has been consistent in older adults [62,63,64]. One possible explanation for the discrepancies between our study and previous studies may be the differences in the study populations. Our study sample included healthy individuals without major comorbidities and non-obese individuals.

This pilot study had some limitations. First, due to its cross-sectional design, causality between body fat distribution and physical performance could not be determined, as sex may have influenced this correlation. Further mechanistic studies are needed to confirm whether a sex-specific role in body fat distribution affects physical performance. Second, our study sample included healthy individuals recruited from a research setting, and our results may not be generalizable to other settings and populations. Third, this study did not adjust for relevant potential confounders, such as independent effects of different fat distributions on physical performance. Fourth, this pilot study may have had limited statistical power owing to the small sample size. Further exploratory studies are needed to assess the influence of body fat distribution on physical performance in a larger study population. Finally, we assessed in vivo muscle fat infiltration with MRI and ^1^H-MRS rather than muscle biopsy samples for this study. However, in vivo ^1^H-MRS is a quantitative method that noninvasively assesses EMCL and IMCL in humans [22,23]. Nonetheless, the major strength of our pilot study was that we performed sex-specific analyses of healthy older men and women matched by age and BMI to assess the correlations of different fat deposits such as whole-body, regional, and cellular adiposity with various physical performance tests. To the best of our knowledge, this is the first study to find a correlation between IMCL content and physical performance, suggesting that muscle-specific calf IMCL content may exert sex-specific effects on physical performance in older adults.

## 5. Conclusions and Implications

Our pilot study showed an inverse correlation between IMCL content and physical performance in healthy older individuals and muscle-specific calf IMCL content based on sex-specific differences. Furthermore, our results suggest that greater EMCL content in the soleus and calf subcutaneous fat might affect physical performance positively in women but not in men. Our findings have practical implications for the prevention of physical disability in older adults that warrant targeting age-related body composition changes with a sex-specific emphasis. The sex-based nature of these relationships may aid in developing more effective interventions to optimize body composition with aging in community health care settings.

## Figures and Tables

**Table 1:** Clinical characteristics of the study subjects.

Variable	Men		Women		*p* Value
	*n* = 20		*n* = 20		
**Clinical characteristics**					
Age, years	78.0	[76.0–80.4]	76.6	[72.7–78.5]	0.072
Height, cm	163.3	[161.6–165.0]	150.3	[147.6–155.0]	0.000
Weight, kg	65.2	[62.0–75.0]	54.7	[51.6–64.6]	0.000
Body mass index, kg/m^2^	24.1	[22.9–27.2]	24.2	[21.9–28.0]	0.841
Waist circumference, cm	86.0	[82.5–91.5]	79.0	[75.0–84.4]	0.005
Hip circumference, cm	91.0	[88.8–95.7]	90.5	[87.9–92.4]	0.414
Waist-to-hip ratio	0.94	[0.91–0.98]	0.89	[0.83–0.93]	0.002
Thigh circumference, cm	44.5	[42.3–48.7]	43.0	[41.1–45.8]	0.121
Calf circumference, cm	34.8	[32.5–36.0]	31.8	[31.0–33.4]	0.014
Physical activity level (metabolic equivalent, minutes/week)	2196	[720–3900]	940	[420–2420]	0.221
Current smokers, %	1	(5.0)	1	(5.0)	0.756
Chronic health conditions					
Hypertension, %	11	(55.0)	16	(80.0)	0.088
Lung disease, %	1	(5.0)	3	(15.0)	0.302
Biochemical parameters in blood samples					
Total cholesterol, mg/dL	165.5	[154.0–203.0]	170.0	[157.5–210.0]	0.620
High-density lipoprotein cholesterol, mg/dL	48.5	[39.3–55.8]	65.5	[53.3–79.5]	0.001
Low-density lipoprotein cholesterol, mg/dL	104.0	[98.5–133.8]	101.0	[76.0–129.8]	0.529
Triglyceride, mg/dL	113.5	[80.3–183.5]	101.0	[86.8–124.5]	0.341
Fasting glucose, mg/dL	99.0	[94.3–103.0]	106.5	[93.0–103.0]	0.495
Fasting insulin, uU/mL	7.7	[4.4–12.8]	7.0	[5.9–9.3]	0.883
Glycosylated hemoglobin, %	5.9	[5.4–6.2]	5.9	[5.7–6.1]	0.529
Creatinine, mg/dL	0.93	[0.83–1.03]	0.69	[0.64–0.79]	0.001
Albumin, g/dL	4.3	[4.1–4.5]	4.4	[4.4–4.5]	0.040
High-sensitivity C-reactive protein, mg/L	0.65	[0.41–2.16]	0.49	[0.35–0.87]	0.270
25-hydroxy vitamin D, ng/mL	19.8	[16.7–27.8]	15.2	[12.1–24.6]	0.134
Physical performance					
Grip strength, kg	34.4	[29.4–35.8]	19.6	[18.3–22.5]	0.001
4-m usual gait speed, m/s	1.13	[1.04–1.28]	1.11	[1.01–1.19]	0.301
Five-times sit-to-stand test, s	8.17	[6.88–9.92]	9.07	[7.89–10.10]	0.120
Timed up-and-go test, s	9.54	[8.43–10.58]	9.92	[9.02–10.58]	0.134
Short Physical Performance Battery, score	12.0	[11.0–12.0]	11.5	[10.0–12.0]	0.242

Values are presented as median (interquartile range) or number (%). *p* values are based on the chi-square, Fisher’s exact, or Mann–Whitney U test.

**Table 2:** Relationship between physical performance and body composition measures in men.

Variable	Grip Strength, kg		Usual Gait Speed, m/s		Five-Times Sit-to-Stand Test, s		Timed up-and-Go Test, s		SPPB, Score	
	r_s_	*p* Value	r_s_	*p* Value	r_s_	*p* Value	r_s_	*p* Value	r_s_	*p* Value
**Dual-energy X-ray absorptiometry parameters**										
Whole-body mass measurement										
Fat mass, kg	−0.245	0.298	0.003	0.990	0.259	0.271	0.126	0.596	−0.004	0.988
Lean mass, kg	0.498	0.026 *	0.194	0.412	−0.165	0.486	−0.212	0.369	0.056	0.815
Percentage of fat mass, %	−0.483	0.031 *	−0.100	0.675	0.269	0.251	0.192	0.416	−0.030	0.901
Segmental body mass measurement										
Trunk fat mass, kg	−0.310	0.184	−0.019	0.937	0.248	0.292	0.167	0.482	0.017	0.944
Appendicular fat mass, kg	−0.146	0.539	0.111	0.642	0.081	0.734	−0.072	0.762	−0.097	0.684
Right lean fat mass, kg	−0.146	0.539	0.134	0.573	0.111	0.640	0.003	0.990	−0.142	0.944
Trunk lean mass kg	0.418	0.067	0.230	0.390	−0.263	0.262	−0.173	0.466	0.073	0.760
Appendicular lean mass, kg	0.586	0.007 **	0.144	0.546	0.009	0.970	−0.180	0.446	−0.037	0.876
Right leg lean mass, kg	0.532	0.016 *	0.157	0.508	0.006	0.980	−0.268	0.254	−0.022	0.925
**Magnetic resonance imaging parameters**										
Calf muscle CSA, cm^2^	0.417	0.068 ^†^	0.287	0.219	−0.253	0.283	−0.415	0.069 ^†^	0.140	0.556
Calf subcutaneous fat CSA, cm^2^	−0.313	0.179	0.002	0.992	0.036	0.880	0.023	0.925	−0.095	0.690
Calf intermuscular fat CSA, cm^2^	−0.041	0.865	0.337	0.146	−0.553	0.011 *	−0.340	0.143	0.021	0.932
Calf intramuscular fat CSA, cm^2^	0.081	0.734	−0.311	0.182	0.552	0.012 *	0.429	0.059 ^†^	−0.500	0.025 *
**Magnetic resonance spectroscopy parameters**										
TA IMCL, mmol/kg wet weight	−0.251	0.300	−0.320	0.182	0.518	0.023 *	0.367	0.123	−0.168	0.492
TA EMCL, mmol/kg wet weight	−0.202	0.408	−0.227	0.351	0.344	0.149	0.284	0.238	−0.125	0.610
SOL IMCL, mmol/kg wet weight	0.404	0.107	−0.026	0.922	0.113	0.667	0.203	0.434	−0.109	0.677
SOL EMCL, mmol/kg wet weight	0.033	0.892	0.038	0.878	−0.018	0.943	−0.040	0.870	−0.033	0.894

r_s_: Spearman’s rank correlation coefficient. ^†^ *p* < 0.10, * *p* < 0.05, ** *p* < 0.01. Abbreviations: SPPB, Short Physical Performance Battery; CSA, cross-sectional area; TA, tibialis anterior; IMCL, intramyocellular lipid, EMCL, extramyocellular lipid.

**Table 3:** Relationship between physical performance and body composition measures in women.

Variable	Grip Strength, kg		Usual Gait Speed, m/s		Five-Times Sit-to-Stand Test, s		Timed up-and-Go Test, s		SPPB, Score	
	r_s_	*p* Value	r_s_	*p* Value	r_s_	*p* Value	r_s_	*p* Value	r_s_	*p* Value
**Dual-energy X-ray absorptiometry parameters**										
Whole-body mass measurement										
Fat mass, kg	0.300	0.199	0.264	0.262	−0.086	0.726	−0.048	0.840	0.278	0.235
Lean mass, kg	**0.467**	0.038 *	−0.050	0.833	−0.100	0.684	0.083	0.729	−0.065	0.784
Percentage of fat mass, %	0.002	0.992	0.405	0.076 ^†^	−0.200	0.412	−0.266	0.257	0.470	0.036 *
Segmental body mass measurement										
Trunk fat mass, kg	0.163	0.493	0.178	0.452	−0.019	0.937	0.045	0.850	0.180	0.447
Appendicular fat mass, kg	0.371	0.107	0.276	0.238	−0.296	0.218	−0.343	0.139	0.500	0.025 *
Right leg fat mass, kg	0.437	0.054 ^†^	0.250	0.288	−0.275	0.218	−0.338	0.145	0.462	0.040 *
Trunk lean mass, kg	0.406	0.076 ^†^	−0.248	0.291	0.111	0.652	0.245	0.298	−0.335	0.149
Appendicular lean mass, kg	**0.505**	0.023 *	0.166	0.485	−0.267	0.270	−0.048	0.840	0.190	0.423
Right leg lean mass, kg	0.388	0.091	0.239	0.311	−0.289	0.229	−0.104	0.663	0.237	0.315
**Magnetic resonance imaging parameters**										
Calf muscle CSA, cm^2^	0.267	0.255	−0.194	0.412	−0.046	0.853	0.141	0.552	0.091	0.704
Calf subcutaneous fat CSA, cm^2^	0.150	0.528	0.447	0.048 *	−0.411	0.081 ^†^	−0.394	0.086 ^†^	0.439	0.053 ^†^
Calf intermuscular fat CSA, cm^2^	0.272	0.182	−0.010	0.967	−0.207	0.395	−0.248	0.292	0.352	0.128
Calf intramuscular fat CSA, cm^2^	0.167	0.481	0.161	0.497	−0.053	0.831	0.021	0.930	−0.048	0.839
**Magnetic resonance spectroscopy parameters**										
TA IMCL, mmol/kg wet weight	0.264	0.276	0.001	0.997	−0.044	0.861	−0.039	0.875	−0.022	0.929
TA EMCL, mmol/kg wet weight	0.433	0.064 ^†^	−0.068	0.783	0.100	0.693	0.072	0.770	−0.192	0.431
SOL IMCL, mmol/kg wet weight	0.160	0.525	0.231	0.356	0.132	0.613	0.472	0.048 *	0.079	0.756
SOL EMCL, mmol/kg wet weight	0.129	0.598	−0.031	0.901	−0.488	0.040 *	−0.337	0.158	−0.180	0.461

r_s_: Spearman’s rank correlation coefficient. ^†^ *p* < 0.10, * *p* < 0.05, ** *p* < 0.01. CSA, cross-sectional area; EMCL, extramyocellular lipid; IMCL, intramyocellular lipid, SPPB, Short Physical Performance Battery; TA, tibialis anterior.

## Data Availability

The data presented in this study are available upon request from the corresponding author. The data are not publicly available due to privacy reasons.
